# The Use of Atypical Antipsychotics in Treating a Pediatric Psychiatric Patient

**DOI:** 10.7759/cureus.27594

**Published:** 2022-08-02

**Authors:** Joshua A Jogie, Kinna Parikh, Sheena Mathew, Kruthiga Rajasekaran, Shaista Arain

**Affiliations:** 1 Department of Neonatology, Port-of-Spain General Hospital, Port-of-Spain, TTO; 2 Research, The University of the West Indies, St. Augustine Campus, Faculty of Medicine, St. Augustine, TTO; 3 Research, GMERS Medical College, Gandhinagar, IND; 4 Research, Government Medical College, Kottayam, IND; 5 Research, Rajah Muthiah Medical College and Hospital, Chidambaram, IND; 6 Department of Pediatrics, University of Kentucky Children's Hospital, Lexington, USA

**Keywords:** atypical antipsychotics, olanzapine, pediatrics, psychiatry, plan

## Abstract

Approaches to enhancing mental health management entail several perspectives and efforts to promote competent treatment. In light of this, we present a case report to describe the nature of events encountered during the management of a psychiatric patient. The paper commences by providing a general introduction and background of the concept of atypical antipsychotics before adding a thesis statement that healthcare providers should be knowledgeable regarding psychopharmacotherapy to effectively design and implement safe patient care. The paper's method involved the review of a case scenario and discussion of the concepts using evidence-based guidance and perspectives. In the case scenario, a pediatric patient significantly gains weight and develops extrapyramidal effects like dystonias, and erratic, jerky neck movements as a consequence of treatment with olanzapine. The most effective care plan involves stopping the medication, reviewing treatment options, and incorporating physical exercise. Most importantly, the plan encourages achieving an adequate heart rate above 100 beats per minute to maintain sufficient perfusion during exercise. The paper concludes by summarizing the perspectives from the studies reviewed.

## Introduction

Quality improvement in psychiatric management often involves several change processes toward establishing a safe and effective healthcare system. Every step in adapting new methods needs to employ the best evidence available as a guiding framework toward change implementation. Daily health interventions and treatments require patient examination and collection of data about their conditions. These approaches invite competent communication ventures and information exchange strategies. Ideally, the current knowledge and evidence require effective communication between healthcare providers and their respective patients [1]. Effective communication and review of treatment approaches may assist in identifying medication side effects and inform the outcome of patient management.

Psychiatric treatments often involve the use of agents that may influence functioning at a higher level. Specifically, therapies involving olanzapine may affect factors related to nutrition, brain motor responses, and tremors and lead to extrapyramidal effects. The drug is a known second-generation antipsychotic that is associated with significant dose-dependent adverse treatment events [2]. Other agents with the same classification include clozapine, quetiapine, risperidone, and asenapine [3]. Among the atypical antipsychotics, olanzapine has a far more significant therapeutic effect compared to many other agents in the same group [4,5]. According to Leucht et al., improvement in performance also means that the agent also has far more serious unwanted effects [6].

Against this background, we present a case report to detail the ideal case management of adverse drug events in the treatment of psychiatric disorders. The treatment with olanzapine and other psychiatric medications may contribute to a more stable thought process and elimination of psychosis and suicidal ideations. However, unregulated utilization of the drugs could lead to the progression of extrapyramidal side effects [7]. Therefore, healthcare providers should be knowledgeable regarding the advanced events in psychopharmacotherapy to effectively plan care approaches and protect the patients from harmful experiences.

## Case presentation

Patient X arrived at the emergency department for consultation with an MD after an initial referral by their primary care physician. The main aim of the session was to review their medication before deciding to continue with these drugs. Regarding the chief complaint, the patient mentioned disturbing nervousness and generalized complaints. The accompanying nurse explained that the patient's symptoms were consistent with general nervousness when in anxiety-inducing situations like attending school and taking on doctor's appointments. The subjective data revealed that the patient experienced jerky erratic tics and a general raising of shoulders when in a tense situation. These movements were mostly involuntary in general, occurring without the patient's awareness. Regarding the history of present illness, the doctor observed the patient having the tics before suggesting that they get a suitable review of treatment before receiving a repeat prescription of their psychiatric treatment medicines. The patient had no known allergies to foods or any medications. An assessment of the previous therapy showed that the patient was on five different regimens for attention deficit hyperactivity disorder and major depressive disorder. These drugs included daily 2.5 mg of olanzapine, 20 mg of amphetamine, fluoxetine at a capsular dose of 20 mg, 25 mg of oral hydroxyzine pamoate, and 10 mg tablets of loratadine once every day.

On assessing the patient's objective data, they had an initial weight of 98.43 kg with a height of 154.9 centimeters. With a BMI of 41, the patient belonged to the 99.5th percentile. Other vitals were as follows: blood pressure of 125/95 mmHg, heart rate of 104 beats per minute, temperature of 36.4 °C, and respiration rate of 20 breaths per minute. The physical examination involving a head-to-toe assessment did not identify any abnormal findings.

Finally, the plan of care required an aggressive approach to stop the adverse drug reactions. The management prioritized stopping olanzapine immediately to reduce significant neuronal harm to the patient. The plan also involved eliminating excessive carbohydrates and prioritizing vegetables with limited protein topping to enrich the taste. Also, the treatment approach advocated for a low to moderate-impact physical exercise that would assist in maintaining the heart rate above 100 beats per minute. The specific activities included cycling and walking. Eventually, pediatric review and possible change of medication were deemed pivotal for the patient's treatment continuity.

The assessment of the patient elicited two critical impressions. The initial impression was that the patient was obese and required a nutritional review. The second impression was that the patient was suffering from the primary side effects of the drug treatment.

## Discussion

Second-generation antipsychotics are serotonin-dopamine antagonists, also identifiable as atypical antipsychotics [8,9]. Multicenter trials to describe the efficacy and suitability of olanzapine have documented that the pharmacological agent is a novel treatment approach for managing psychotic symptoms and schizophrenia [10,11]. According to various studies in the literature, olanzapine use has drastically decreased over the years because of the associated weight gain and adverse metabolic side effects. These factors show that the patient's weight gain was primarily due to unchecked drug consumption. With a BMI of 41, the patient most definitely had a weakened immune system and high susceptibility to infections.

Regarding the drug's mechanisms of action, these agents are primarily D2 antagonists, whereas additional properties may include 5-HT2A antagonism and 5-HT1A agonism [12]. Though more effective compared to other atypical antipsychotics on average, olanzapine's side effects may be relatively more severe compared to those of other second-generation antipsychotics [13,14]. In our case, the patient experienced significant treatment adverse events. They developed ticks characterized by erratic solid jerky movements in the neck region. According to D'Souza and Hooten, the exact extrapyramidal symptom spectrum may include dystonias, akathisia, and parkinsonism [15]. The patient's condition may be attributed to the antagonistic binding of D2 receptors in the brain's mesolimbic and mesocortical pathways. Toxicity management requires a therapeutic adjustment of doses based on the reviews provided by the National Center for Biotechnology [16]. Advanced patient treatment of obesity involves adopting exercise to improve metabolism and encourage caloric burn. Eventually, the patient's weight showed a significant reduction in the post-interventional phase based on the milestone visits and progress documentation. The current evidence, therefore, recommends dose adjustment and a review of treatment options for patients who experience olanzapine toxicity.

## Conclusions

Psychiatric practice requires adequate and in-depth knowledge of psychopharmacotherapy and related adverse events to effectively plan the safe treatment of patients. This report highlights the importance of adopting ethical perspectives in decision-making and prioritizing patients' wellbeing. We did not mention the name of the patient in the report. Additionally, the priority in managing the condition was to ascertain the patient's wellbeing. Stopping the treatment at once assisted in limiting future and continued chemotoxicity. During the patient's medical reconciliation, additional efforts are essential in ensuring the management of weight gain. The evidence-based perspectives suggest that the patient should engage in low to moderate-intensity exercise. Additionally, the strategy should ensure that the heart rate constantly remains above 100 beats per minute to maintain adequate perfusion. Finally, we refrained from revealing personal details of the patient to the public, adhering to the principles of privacy and patient confidentiality.
